# Optimisation and Benchmarking of Targeted Amplicon Sequencing for Mycobiome Analysis of Respiratory Specimens

**DOI:** 10.3390/ijms20204991

**Published:** 2019-10-09

**Authors:** Nur A’tikah Binte Mohamed Ali, Micheál Mac Aogáin, Raika Francesca Morales, Pei Yee Tiew, Sanjay H. Chotirmall

**Affiliations:** 1 Lee Kong Chian School of Medicine, Nanyang Technological University, 11 Mandalay Road, Singapore 308232, Singapore; 2 Department of Respiratory and Critical Care Medicine, Singapore General Hospital, Singapore 169608, Singapore

**Keywords:** mycobiome, targeted amplicon sequencing, ITS, respiratory microbiome

## Abstract

(1) Background: Firm consensus has yet to be established in relation to taxonomic classification and primer choice in targeted amplicon sequencing of the mycobiome. While the nuclear ribosomal internal transcribed spacer (ITS) region are recognized as the formal fungal taxonomic barcode, appraisal of different ITS sub-regions and the influence of DNA extraction methods have not been comprehensively undertaken using human respiratory specimens. (2) Methods: We performed ITS analysis of respiratory (sputum) samples by assessing (a) the effect of alternate DNA extraction techniques and (b) an evaluation of four different ITS primer pairs (ITS1F and ITS2; ITS1-30F and ITS1-217R; gITS7ngs and ITS4ng; and Fseq and Rseq) on the mycobiome profiles generated for mock fungal communities and their respective clinical (airway) specimens. (3) Results: Primer pairs varied in their resulting ITS mycobiome profiles, suggesting that particular pairs may be more relevant for analysis of respiratory samples compared to others. Assessment of DNA extraction methods highlighted lower final DNA concentrations achieved by mechanical disruption compared to enzymatic lysis. However, despite lower yields, DNA liberated by mechanical lysis more readily yielded ITS bands with highest success in combination with the Fseq and Rseq primers. (4) Conclusion: Choice of extraction method, primers used, and sequencing approach are all important considerations in sequencing the mycobiome and should be tailored to sample type. A standardization of approach to mycobiome studies using respiratory specimens will permit more reliable comparisons between studies and improve our understanding of the role of fungi in the human airway.

## 1. Introduction

Fungal disease affects more than 300 million people worldwide, accounting for an estimated 1.5 million deaths annually [1]. Although affecting almost every organ system, the respiratory tract is an important portal for fungal access to the human lungs, particularly environmentally abundant and ubiquitous fungal spores that enter by inhalation and, under the right conditions, establish infection [2]. While phagocytic and mucociliary clearance are employed effectively in healthy non-immunocompromised individuals, those with chronic respiratory diseases such as chronic obstructive pulmonary disease (COPD) or bronchiectasis have anatomically abnormal airways and dysfunctional immunity. This results in higher risks of fungal acquisition, colonization, and potential complications [2].

High throughput sequencing (HTS) of the airway has uncovered novel associations between the pulmonary microbiome and chronic respiratory disease [3]. To date, most literature on the pulmonary microbiome has a bacterial focus [4,5]. In contrast, the sequencing of the fungal microbiome (the mycobiome) has significantly lagged behind and is applied in relatively few studies [6,7,8,9]. Targeted amplicon sequencing is used in most published work focused on the mycobiome. This contrasts shotgun sequencing, which is hindered by the greater relative abundance of bacterial DNA to that of fungi in clinical samples [10]. However, unlike bacterial 16S rRNA bacterial sequencing, consensus has yet to be reached in relation to a universal protocol for internal transcribed spacer (ITS) sequencing [10]. Further, the handling of currently available ITS amplicon sequencing protocols on different clinical samples has not been systematically investigated and the optimal primers and conditions for application on airway specimens (sputum) is not established. Careful consideration of primer selection is crucial and key factors include coverage, amplicon length, taxonomic resolution, and accuracy [11]. While the internally transcribed spacer region (ITS) is recognized as a universal taxonomic barcode for fungi, appraisal of different ITS subregions and the influence of DNA extraction methods have not been comprehensively examined using respiratory specimens, making it challenging to compare results between existing respiratory mycobiome studies, both between disease states and in different patient cohorts [12,13,14,15].

Although ITS1 primers (i.e., ITS1F and ITS2) have been employed in many large-scale projects, recent findings demonstrate that these primers suffer from primer bias and amplification bias and fail to fully capture predetermined fungal mock community profiles, leading to inaccuracies in mycobiome evaluation [12,13,14,15]. Consequently, the development of alternative primer sets has accelerated lately. The development of novel ITS primers such as ITS1-30F and ITS1-217R illustrate improved taxonomic coverage and read recovery. However, they have only been assessed in cervicovaginal, anal, and oral mouthwash samples [16]. The current literature suggests a preference for targeting the ITS2 region (over ITS1) as it possesses a more universal primer site and lower length variation, leading to less taxonomic bias [17,18,19]. Nilsson and colleagues suggested targeting ITS2 subregions by using gITS7ngs and ITS4ng based on a superior coverage of the fungal kingdom [13]. However, its accurate taxonomic resolution has yet to be evaluated experimentally, particularly in clinical respiratory samples [13]. McTaggart recently examined the respiratory mycobiome profile in broncho-alveolar lavage (BAL) specimens, known to be culture-positive or -negative for *Blastomyces*. They compared a range of primers targeting different ITS regions, representing an important benchmarking study for application on other respiratory disease states or specimen types such as sputum [11].

Further studies are clearly required if the mycobiome field is to advance. Reaching a methodological consensus for different anatomical sites would be an excellent start, similar to that described for bacterial 16S rRNA sequencing. Here, we present data on the ITS analysis of respiratory (sputum) samples by assessing (a) the effect of alternate DNA extraction techniques and (b) an evaluation of four different ITS primer pairs (ITS1F and ITS2; ITS1-30F and ITS1-217R; gITS7ngs and ITS4ng; and Fseq and Rseq) on the mycobiome profiles generated for mock fungal communities and their respective clinical (airway) specimens using the MiSeq platform and a targeted ITS sequencing workflow.

## 2. Results

### 2.1. Detection of Specific Fungal Taxa by ITS Sequencing

**Figure 1:**
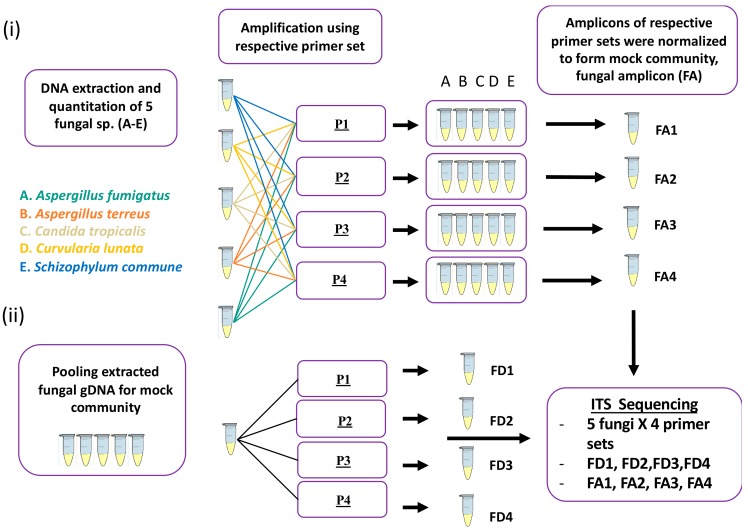
Schematic overview of ITS amplicon generation and mock community preparation method. The two strategies for preparation of mock communities are outlined including (**i**) fungal amplicon method; FA: ITS amplicons were generated from DNA isolated from individual strains using all the four primer sets and resultant amplicons were normalized based on copy numbers and pooled after purification and quantification. Each mock community contained five fungal species. (**ii**) Fungal DNA method; FD: DNA from fungal isolates (Table 1) was extracted, quantified, normalized, and pooled at the same concentration, and the pool was then used as a DNA template for amplification using all four primer sets (P1–P4, Table 2).

**Figure 2:**
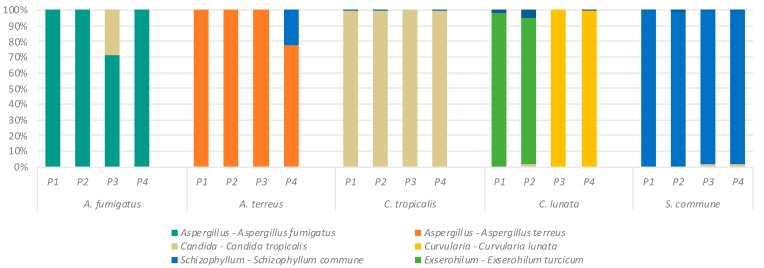
Analysis of classified ITS sequences derived from pure fungal cultures using different primer sets. Colored bars indicate the percentage of ITS reads mapped to indicated taxa; *Aspergillus* (*A. fumigatus/A terreus*), *Candida* (*C. tropicalis*), *Curvularia* (*C. lunata*), *Schizophyllum* (*S. commune*), and *Exserohilum* (*E. turcium*).

We first assessed the ability of our sequencing workflow to correctly classify fungi of known identity (Table 1) using four established ITS primer sets (Table 2). Following extraction of DNA from all fungal isolates using the Roche PCR pure template kit, primer sets P1–P4 were used to amplify the ITS regions. The resultant amplicons, derived from the isolated template DNA, were then used to create amplicon sequencing libraries and sequenced to confirm the identity of each amplicon (Figure 1). Results are shown in Figure 2 and highlight the broad agreement of amplicon classification with each correctly identified fugal isolate. One exception is the identification of *Exserohilum* sp., using primer sets P1 and P2 for DNA extracts from *Curvularia lunata.* In contrast, primer sets P3 and P4 correctly identified *Curvularia* and *C. lunata* as the fungal species present in the test sample.

### 2.2. Effect of Primer Selection on Recovery of Fungal Mock Community Structure

**Figure 3:**
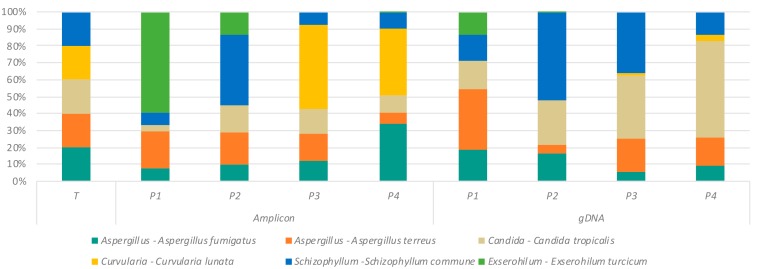
Performance of primer sets in replicating the theoretical (T) composition of amplicon and genomic DNA-based mock communities. Mock communities were subjected to amplification of the ITS region using primer sets P1–P4.

Having demonstrated the relative reproducibility of individual fungal identities (Figure 2), we next sought to assess the fidelity of ITS primers in reproducing predetermined fungal mock communities derived from both purified amplicon sequences (FA) and fungal genomic DNA (FD) (Figure 1). Results from ITS sequence analysis using primer sets P1–P4 revealed the composition of the mock community to be generally reproducible but the discrepant results related to primers P1 and P2 and the misidentification of *Curvularia* (as *Exserohilum*) was again noted (Figure 3). This issue was specific to primer sets P1 and P2 only and was not observed in analysis of ITS profiles of mock communities amplified with primer sets P3 and P4. Important differences were additionally observed between amplicon- and DNA-derived mock community profiles: notably *Curvularia/Exserohilum* were less abundant in the gDNA-derived mock community while *Candida* was more readily detected in the gDNA-derived community (Figure 3).

### 2.3. Assessment of DNA Extraction Methods and Their Effect on ITS Amplification

**Figure 4:**
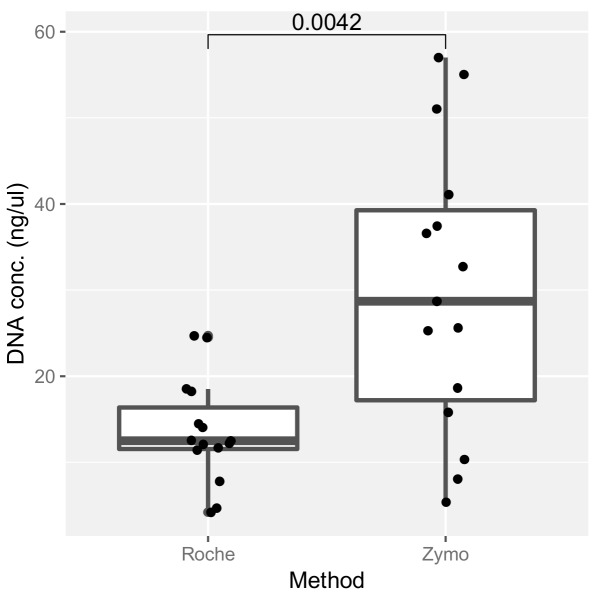
Comparison of DNA yield extracted from clinical (sputum) samples using the Roche and Zymo protocols. Sputum samples were split into two aliquots and subjected to both extraction methods. Resultant DNA was quantified using a qubit fluorometer. The *p*-value for a Mann–Whitney U test of differences between methods is indicated, suggesting greater DNA extraction efficiency of the Zymo kit.

In order to generate data on the mycobiome in clinical samples, positive amplification of the ITS region is first required. As a first step to determining the relative sensitivity of each primer pair in yielding ITS bands from clinical (sputum) samples, we compared the DNA yields achieved using two commercial kits: the Roche High Pure PCR Template Preparation Kit (Roche) and the Quick-DNA™ Miniprep Plus Kit (Zymo Research). Our results demonstrate a higher DNA yield with the Zymo extraction method compared to that of the Roche kit (Figure 4). We next assessed the ability of the different primer sets to generate fungal amplicons on DNA from clinical (sputum) samples, extracted with the different DNA extraction methods (kits). Interestingly, this revealed the superior performance of the Roche DNA extraction kit overall, particularly when used in combination with primer set P4, a combination that led to the successful amplification of an ITS band in greater than 90% of tested sputum samples (Table 3). Therefore, while the Roche kit extraction method provides lower DNA yields, overall quality and success for mycobiome sequencing appears superior with this approach.

### 2.4. Variation in Mycobiome Profiles Derived by Primer Sets P1–P4 Compared to Dual ITS1-ITS2 Targeted Amplicon Shotgun Sequencing

**Figure 5:**
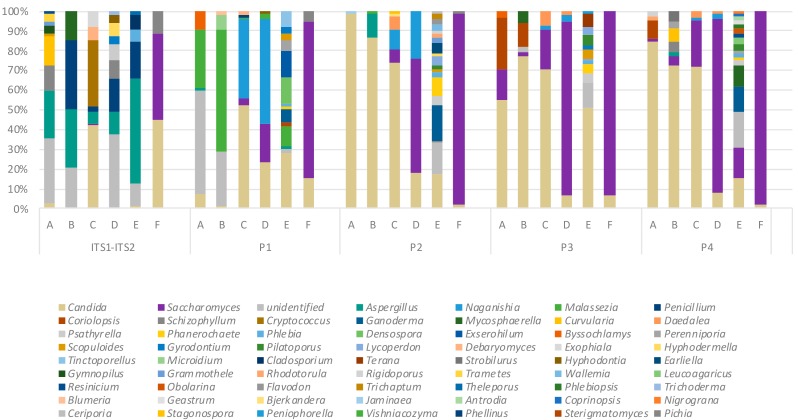
Analysis of sputum mycobiome profiles obtained by alternative sequencing approaches including dual ITS1-ITS2 targeted amplicon shotgun sequencing and ITS1 or ITS2 targeting techniques applied in this study. Colored bars represent relative abundance of the indicated taxa. DNA derived from sputum samples from bronchiectasis patients (A–F) served as a DNA template.

We next applied our sequencing workflow to a collection of sputum samples from patients with bronchiectasis where the mycobiome had been characterized by a dual ITS1-ITS2 targeted amplicon shotgun sequencing approach [9]. This allowed comparison between profiles generated by the P1–P4 primer sets which target either the ITS1 (P1 and P2) or ITS2 (P3 and P4) regions. The resulting mycobiome profiles exhibited marked differences across primer pairs and sequencing strategies with a greater abundance of *Aspergillus* sp. identified in the ITS1-ITS2 amplicon profiles (Figure 5). ITS1-targeting primer pair P1 tended to amplify more *Malassezia* sp. while there was an increasing abundance of *Candida* and *Saccharomyces* sp. observed in profiles generated with primer sets P2–P4 (Figure 5). Somewhat surprisingly, several mushroom, corticioid, polypore, and soil-associated taxa were identified. These likely represent transient indoor fungal propagules that may exhibit dynamic trafficking between lung and environment rather than existing as true constituents of the lung mycobiome. [25] Indeed such taxa, though detectable by PCR, may not be viable. Taken together, this illustrates that the diversity of observed fungal taxa is dependent on primer choice and sequencing method and includes both resident fungi as well as diverse transient environmental propagules of the exposome. Fungal diversity and that of their nutritional modes is summarized by reference to FUNGuild database as illustrated in Supplementary Table S1 [26]. In spite of such richness, the true extent of fungal diversity and their nutritional modes in the environment is likely underestimated and may be even greater than that observed here. [27]

### 2.5. Key Variables in Determining ITS Mycobiome Profiles of Clinical Respiratory Samples

**Figure 6:**
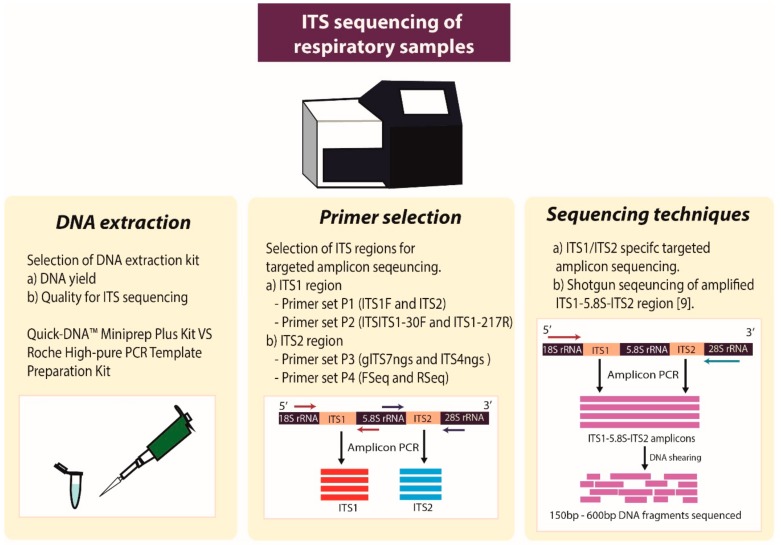
Schematic overview of important considerations for ITS sequencing of respiratory specimens, based on the findings of this study. DNA extraction methods may yield variable results in terms of yield and capacity to amplify the ITS region. Primer selection can influence the outcome significantly, both by determining the chances of successful ITS amplification and in terms of resultant ITS profiles derived. Our findings suggest the optimal pairing of the Roche extraction methods with ITS primer pair P4. Finally, the choice of amplicon sequencing technique may also contribute to differences in the final derived ITS mycobiome profiles.

Our work has identified a number of important factors that influence the mycobiome profiles generated in targeted analysis of the ITS regions. These include the DNA extraction methodology employed, the chosen primer pair, and the different sequencing strategies adopted, all of which can have a bearing on the resultant mycobiome profiles observed in clinical studies (Figure 6).

## 3. Discussion

The microbiome has been investigated in respiratory disease, with the mycobiome more recently emerging as an important area of research [9,28,29]. While there is much interest in the mycobiome from a medical standpoint, its assessment brings several challenges compared to the bacterial microbiome and is therefore less often investigated in respiratory studies [11,30]. Culture-independent tools to characterize and profile the mycobiome therefore lack standardization.

In this study we sought to define the utility of several primer sets that target the ITS1 or ITS2 regions of the fungal 18S–28S intergenic regions, in the characterization of the human airway (sputum) mycobiome. We present data on the ITS analysis of sputum samples by assessing (a) the effect of alternate DNA extraction techniques and (b) an evaluation of four different ITS primer pairs (P1, P2, P3, and P4) on the mycobiome profiles generated for identified fungal isolates, mock fungal communities, and clinical respiratory (sputum) specimens. While our initial analysis yielded largely consistent findings in relation to the identification of pure fungal cultures, we did identify the apparent misidentification of *C. lunata* as *Exserohilum* sp. while using primer sets P1 (ITS1F, ITS2) and P2 (ITS1-30F, ITS1-217R). Though these primarily phytopathogenic species do not represent major lung pathogens, the misidentification nonetheless highlights the potential for misclassification when different primer sets are used. In contrast, for primer sets P3 (gITS7ngs, ITS4ngs) and P4 (Fseq, Rseq), no such ambiguity was observed, and *Curvularia* was correctly identified. This suggests higher accuracy associated with primers targeting ITS2, a region which is suggested to be more appropriate than ITS1 as a taxonomic marker in terms of lower size variability and associated taxonomic bias [13]. In this case, greater variability in the ITS2 region of the *Exserohilum turcicum* reference sequence (SH528234.07FU) relative to that of *Curvularia lunata* (SH187568.07FU) allowed more accurate identification compared with primers targeting the ITS1 regions, which share high identity in these taxa. This classification discrepancy was also observed for P1 and P2 in our mock community analysis for these primer sets only. An interesting observation to emerge from our mock community analysis was the discrepancy between our amplicon-generated mock community and our genomic DNA-generated pool. These differences likely reflect the precision with which amplicon fragment copy numbers can be determined relative to genomic DNA which may contain multiple copies of the ribosomal genes. This is also an important consideration for the mycobiome analysis of clinical samples where taxa with many ribosomal copies may be overestimated. A further consideration is PCR bias which favors amplification of smaller ITS regions such as in the case of particular *Candida* species [31]. Such biases may be mitigated to some extent by choosing to target the ITS2 region, which exhibits less size variation in addition to greater taxonomic specificity relative to ITS1 while circumventing known biases associated with ITS1 primer sets [22,32,33]. However, this still remains a limitation of targeted amplicon sequencing which must be given due consideration. Long-read sequencing (PacBio) represents an alternative approach that does not suffer from this bias to the same degree [34].

To the best of our knowledge, aside from primer set P1, no literature has to date evaluated the fungal profiling of the different primer sets (P2, P3, and P4) on clinical samples and mock fungal communities. Primer sets P2 and P4 have been used to assess the mycobiome profiles of fecal, cervicovaginal, anal, and oral mouthwash samples, whereas primer set P3 has yet to be evaluated experimentally [13,16,23,24,35]. As the amplification of ITS sequences is a prerequisite to generation of fungal mycobiome data from clinical specimens, we sought to investigate under which conditions amplification was favored in clinical respiratory (sputum) samples. We conclude that primer set P4, in combination with the Roche extraction kit and our bead-beating protocol, had the highest success rate in getting positive amplification from sputum. Interestingly, this particular approach, which yielded the highest success rate, was surprising given the observation that the Zymo enzymatic lysis kit generated higher DNA yields. This suggests that DNA liberated from the Zymo kit is not as amenable to ITS amplification, possibly due to the presence of inhibitors or preferential extraction of DNA from other nonfungal sources including bacterial and/or human cells, both likely to be abundant in clinical respiratory (sputum) samples. Importantly, while total DNA yield is high with both approaches, the fungal DNA component is lower, possibly because of the need for additional bead beating, which helps to lyse fungal cell walls that would otherwise remain intact and hinder DNA release [36,37]. Our overall conclusion is that the use of bead beading with subsequent clean-up using the Roche protocol and amplification of the ITS2 region with primer pair P4 appears an optimal selection for working with respiratory (sputum) samples in these patients due to greater sensitivity in the amplification of the fungal ITS region. However, further head-to-head comparisons across a broader range of fungal species and respiratory sample types (e.g., broncho-alveolar lavage (BAL)) should be performed.

Finally, we sought to compare the P1–P4-generated sputum mycobiome profiles against profiles generated by targeted amplicon shotgun sequencing of the ITS1-ITS2 region in bronchiectasis patients [9]. This uncovered several interesting findings. Firstly, several differences between patients assessed by different primer sets were observed, including a higher abundance of *Aspergillus* in the ITS1-ITS2 generated groups and a tendency toward more *Malassezia* in P1, and both *Candida* and *Saccharomyces* in P2–P4. This highlights the profound effect that primer and marker selection can have on resultant reported mycobiome profiles in addition to other factors including DNA extraction and sequencing techniques (Figure 6). While the ITS1-ITS2 amplicon shotgun methods could be said to have identified the more clinically relevant pathogens including *Aspergillus*, *Cryptococcus*, and *Penicillium*, this method is also more operationally complex and expensive to employ, requiring careful shearing and size selection of long ITS1-ITS2 spanning DNA fragments, which is not as streamlined as current methods for sequencing either ITS1 or ITS2 alone [38]. While we based our analysis around a single analytical framework, it is important to note that the choice of analytical pipeline can further influence derived fungal profiles and is a major consideration in mycobiome research requiring international collaboration and alignment given the diversity of wet- and dry-lab protocols currently adopted [39,40]. Determining which profiling method is truly best will require additional work and a direct head-to-head comparison of the different methodologies in larger clinical studies to further probe the precision with which each method can predict clinically important phenotypes. This will be aided by further optimization of ITS sequencing protocols, tailoring for specific respiratory clinical specimens and assessing their utility as predictive markers, allowing deeper insight into fungal pathogenesis and fungal–host interaction.

## 4. Materials and Methods

### 4.1. Clinical Samples and Cultured Fungal Isolates

Twenty-one patient sputum samples [COPD (*n* = 15), bronchiectasis (*n* = 6)] were used in this study. Clinical information on the recruited patients is provided in Table 4. At sample collection, an equal volume of Sputasol (Oxoid limited, Basingstoke, Hampshire, England) was added to each specimen and shaken for 15 min at 37 °C. Two volumes of RNAlater (Life technologies, Penrose, Auckland, New Zealand) were then added and the Sputasol-homogenized samples stored at −80 °C until further processing [9]. Ten fungal isolates, obtained from clinical and environmental sources and whose identity was confirmed by morphological analysis and sequence comparison of the β-tubulin (Tub2) gene, RNA polymerase II (RPB2), ITS, and 18S sequences were used in this study (Table 1). Pure cultures were subcultured on Sabouraud Dextrose agar (SDA) at 30 °C for 4–7 days prior to DNA isolation.

### 4.2. DNA Extraction and Quantification

Sputum samples in RNAlater (Life technologies, Penrose, Auckland, New Zealand) were centrifuged at 13,000 rpm for 10 min and 500 μL sterile phosphate-buffered saline PBS (GE Lifesciences, Uppsala county, Sweden) was added to re-suspend resultant pellets. As for fungal cultures, the samples were homogenized through bead beating and the DNA was purified using the Roche High Pure PCR Template Preparation Kit (Hoffmann, La Roche, Basel, Switzerland). In parallel, 200 μL of selected sputum samples in RNAlater (Life technologies, Penrose, Auckland, New Zealand) was used for DNA extraction and purification using the Quick-DNA™ Miniprep Plus Kit (Zymo Research, Irvine, CA, USA) according to the manufacturer’s instructions. All purified DNA was quantified using the Qubit™ fluorometer 2.0 double-stranded DNA (dsDNA) assay (Invitrogen, Carlsbad, CA, USA). The DNA quality was assessed using the NanoDrop™ Spectrophotometer (Thermo Fisher Scientific, Wilmington, DE, USA). Fungal cultures grown on SDA were scraped and mixed with 500 μL PBS. The samples were then transferred to sterile bead mill tubes (VWR) containing 1 mm sterile glass beads (Sigma-Aldrich, Saint Louis, MO, USA). Homogenization using a beads mill homogenizer (VWR international, Montréal, QC, Canada) was then performed followed by DNA purification using the Roche High Pure PCR Template Preparation Kit (Hoffmann, La Roche, Basel, Switzerland).

### 4.3. Mock Community Preparation

Two types of mock community were prepared, referred to as fungal DNA (FD) and fungal Amp (FA) throughout. (i) FD: DNA from fungal isolates (Table 1) was extracted, quantified, normalized, and pooled at the same concentration, and the pool was then used as a DNA template for amplification using all four primer sets (Table 2). (ii) FA: ITS amplicons were generated from DNA isolated from individual strains using all the four primer sets and resultant amplicons were normalized based on copy numbers and pooled after purification and quantification. Each mock community contained five fungal species as listed in Table 1.

### 4.4. PCR Amplification

All primers (Integrated DNA technologies, IDT, Coralville, Iowa, USA) were synthesized with the respective forward and reverse overhang adaptors as described in Illumina ITS metagenomics demonstrated protocols (https://bit.ly/30mczJZ) [38]. For each primer pair, PCR was optimized for annealing temperature using a Verti Thermal (Invitrogen, Carlsbad, CA, USA). Based on these trials, PCR was performed in a 20 μL reaction mixture with 2 μL input of fungal DNA, 10 μL KAPA HiFi HotStart ReadyMix polymerase (Kapa Biosystems, Salt River, Cape Town, South Africa), and 4 μL (1 μM) of each primer (IDT, Coralville, Iowa, USA). Thermocycling was performed on a Verti Thermal cycler (Invitrogen, Carlsbad, CA, USA) and included an initial denaturation of 95 °C for 3 min, followed by 35 cycles of 98 °C for 20 s, 50 °C for 15 s, and 72 °C for 15 s, followed by a final extension of 72 °C for 5 min. Negative controls were included for all PCR batches and for all primer sets. Amplicons were loaded into a 1.5% agarose gel and run at 100 V for 25 min to check for the presence of the bands before proceeding with sequencing library preparation for next-generation sequencing.

### 4.5. Sequencing: Library Preparation and Subsequent Sequencing

Amplicons were purified with Agencourt AMPure XP beads (Beckman Coulter, Brea, CA, USA). Library preparation was performed based on Illumina’s ITS metagenomics demonstrated protocol [2]. Agarose gel electrophoresis was performed on post clean-up of Index PCR products to ensure that the products were successfully purified without the presence of primer dimers before proceeding with quantification and pooling. Pooled denatured libraries were quantified using the KAPA library quantification kit (Kapa Biosystems, Salt River, Cape Town, South Africa). High throughput sequencing of paired end libraries was performed on an Illumina MiSeq (Illumina, San Diego, CA, USA) system running a 2 X 300 bp sequencing protocol. All raw sequence data associated with this study has been uploaded to the sequence read archives (SRA) under project accession PRJNA565350.

### 4.6. Data Analysis

Targeted amplicon sequences were analyzed using the ITS Metagenomics pipeline (version 1.0.1; Basespace labs, San Diego, CA, USA), using as a taxonomic reference database the v7.2 UNITE project described by Köljalg et al. [10]. Control samples from negative PCR and blank DNA extractions were also sequenced and assessed to detect potential contaminants using the decontam statistical package (version 1.4.0) [41].

### 4.7. Ethics

Collection of all clinical samples used in this study was approved by the respective Institutional Review Boards (IRB) as follows: NTU IRB-2016-01-031 (14/03/2016); NTU IRB-2017-03-013 (25/04/2017); CIRB 2017/2933 (31/01/2017); and CIRB 2016/2073 (22/03/2016).

## Figures and Tables

**Table 1:** Fungal species used in this study representing common human respiratory-associated species. Ascomycota (*n* = 4) and Basidiomycota (*n* = 1) were included in mock communities tested to examine for preferential PCR amplification and sequencing outcomes.

Phylum	Fungal Species
Ascomycota	*Aspergillus fumigatus*
	*Aspergillus terreus*
	*Candida tropicalis*
	*Curvularia lunata*
Basidiomycota	*Schizophyllum commune*

**Table 2:** List of selected primer sets used in this study. Primer sets P1 and P2 target the ITS1 region, while primer sets P3 and P4 target the ITS2 region.

Primer Set	Target	Forward Primer (5′ > 3′)	Reverse Primer (3′ > 5′)	References
P1	ITS1	**ITS1F**: CTTGGTCATTTAGAGGAAGTAA	**ITS2**: GCTGCGTTCTTCATCGATGC	[20]
P2	ITS1	**ITS1-30F**: GTCCCTGCCCTTTGTACACA	**ITS1-217R**: TTTCGCTGCGTTCTTCATCG	[16]
P3	ITS2	**gITS7ngs**: GTGARTCATCRARTYTTTG	**ITS4ngs**: TCCTSCGCTTATTGATATGC	[13,21,22]
P4	ITS2	**Fseq**: ATGCCTGTTTGAGCGTC	**Rseq**: CCTACCTGATTTGAGGTC	[23,24]

**Table 3:** Success rate of ITS band amplification from COPD sputum samples (*n* = 15) using different DNA extraction techniques and primer sets. ROCHE: Roche High Pure PCR Template Preparation Kit. ZYMO: Quick-DNA™ Miniprep Plus Kit. P1: ITS1F and ITS2. P2: ITS1-30F and ITS1-217R. P3: gITS7ngs and ITS4ng. P4: Fseq and Rseq.

Method	Primer Set			
	P1	P2	P3	P4
Roche	53%	80%	40%	93%
Zymo	53%	60%	33%	80%

**Table 4:** Demographics of the study population used in this study. All COPD and bronchiectasis patients were recruited in Singapore. Data are presented as mean or median (and standard deviation, SD or interquartile range, IQR) or *n* (percentage; %). Post BD: post bronchodilator; BMI: Body mass index; BSI: Bronchiectasis Severity Index.

Characteristics	COPD Samples (*n* = 15)	Bronchiectasis Samples (*n* = 6)
Age (years): Mean ± SD	69 ± 9.2	64.6 ± 9.2
Gender (male): *n* (%)	14 (93.3)	3 (50.0)
BMI (kg/m2): Mean ± SD	22.2 ± 5.5	22.5 ± 6.1
Smoking history: *n* (%)		
Nonsmoker	0 (0)	3 (50.0)
Current	8 (53.3)	0 (0)
Ex-smoker	7 (46.7)	3 (50.0)
COPD assessment test (CAT): Mean ± SD	17.6 ± 7.9	-
Post BD FEV1 (% predicted): Mean ± SD	58.1 ± 14.5	70.4 ± 24.3
Post BD FEV1/FVC (% predicted): Mean ± SD	51.0 ± 8.4	-
BSI score	-	7 (5–13)
No. of exacerbations in previous year: Median (IQR)	1 (0–2)	0 (0–6)

## Supplementary Materials

Supplementary Table S1: FUNGuild analysis of top 40 ecological guild units identified in clinical specimens. The top 40 OTUs identified in clinical specimens (Figure 5) are indicated according to their closest matched species hypothesis within the UNITE database. Output from the FUNGuild database are indicated including taxon, rank, trophic mode, guild, confidence ranking, growth form, trait notes, and data source/citation.

## Abbreviations

ITS	internally transcribed spacer
COPD	chronic obstructive pulmonary disease
